# The Future of Ageing: not more of the same

**DOI:** 10.1007/s10522-017-9720-x

**Published:** 2017-07-05

**Authors:** Ewa Sikora, Suresh I. S. Rattan

**Affiliations:** 1 Nencki Institute of Experimental Biology, Polish Academy of Sciences, Warsaw, Poland; 2 Department of Molecular Biology and Genetics, Aarhus University, 8000 Aarhus, Denmark

The 10th European Congress of Biogerontology, combined with the 6th Biogerontological Meeting, was held at the Nencki Institute, Warsaw, Poland, 23–24 September, 2016. This special issue of *Biogerontology* is a collection of papers selected from the submitted articles, after proper peer-reviewing, and are representative of 20 invited lectures, six oral presentations and 52 abstracts presented at the congress by the participants from 24 countries worldwide.

The theme of the meeting “The Future of Ageing”, was based on the fact that most of us expect to live a long life in the future, and so will surely experience ageing and old age with all its highs and downs. Understanding the biological, biomedical, demographic and psycho-social aspects of ageing is the necessary road to prepare for such a future. Since a major challenge to consider is that could we, would we, and should we stop ageing partially or completely, all invited speakers were asked to address the following five questions:

1. What have we understood about ageing so far?
2. What are the gaps in our present understanding of ageing?
3. How age-related diseases are connected to the ageing process?
4. Can ageing be postponed?
5. How long should we live?

In keeping up with the theme of the congress, a wide range of topics were covered, and attempts were made to address the above questions. These topics included the evolutionary basis of ageing and longevity, the genetic, epigenetic, cellular and molecular mechanisms of ageing, the new and old experimental models of ageing, life history course of ageing, and how life style and other interventions can influence ageing and longevity. What follows is a brief commentary on the papers published in this special issue.

Croco et al. (2017) have analyzed the possibility that similar cellular mechanisms have been influenced by the forces of natural selection to create a convergent evolution of longevity as suggested by the “recent” acquisition of an extended longevity and large body mass of some species of mammals present on the earth today. They have reviewed the literature, focusing on two cellular features: (1) the capacity for extensive cellular proliferation of differentiated cells, while maintaining genome stability; and (2) the capacity to detect DNA damage. They conclude that, “longevity and body mass are both positively linked to these cellular mechanisms”.

Although gerontology is primarily a human-oriented discipline, a wide variety of experimental model systems are used to dissect the mechanisms of human ageing and longevity. These systems include lower organisms, insects, nematodes, rodents, and cells in culture. For example, normal adult human skin fibroblasts were to show the importance of the non-canonical, telomere-unrelated functions of catalytic subunit of telomerase reverse transcriptase (TERT) in replicative senescence (Yehuda et al. 2017). Similarly, both fibroblasts and keratinocytes have been shown to be useful in dissecting the mechanisms of photoageing and in testing new phytocomponents (Cavinato et al. 2017). Augustyniak et al. (2017) in turn, have used human iPSCs to reveal the role of mitochondria in neural differentiation. As a point of caution about using model systems, Bilinski et al. (2017) argue that, “the object of experimental studies should be animals which are closest relatives of human beings in evolutionary terms, rather than lower organisms, which do not have sufficient complexity in terms of tissues and organ structures”.

Epigenetics and its role in ageing and age-related disease is a focus of review by Gensous et al. (2017). They stress the importance of adopting the conceptual framework of ‘‘geroscience’’, which starts from the observation that advanced age is the major risk factor for several age-related pathologies and aims at identifying the mechanistic links between aging and age-related diseases, which can be an “epigenetics clock”.

Since old age is marked by many disorders, frailty and disability, several articles in this special issue address that. Ageing of the immune system—immunosenescence—and its relevance to age-related cancer is reviewed by Pawelec (2017). Interestingly, it seems that immunosenescence differentially affects women and men as reported in studies conducted to reveal the influence of gender and labour status to some immunological parameters (Dudkowska et al. 2017). Furthermore, the process of immunosenescence is strictly connected with inflammageing, which manifests as an increase level of pro-inflammatory molecules in the elderly in comparison with young people. Bednarska-Makaruk et al. (2017) discuss the pro-inflammatory role of resistin in the development of vascular type dementia.

Frailty seems to be a universal and useful marker for determining the health status of individuals. Mitnitski et al. (2017) present their network model of health deficits to better understand how changes in health are captured by the frailty index, FI. Similarly, the condition of the elderly is measurable by using simple tests, such as Up-and Go test, which can be very useful for assessing the muscle strength (Zarzeczny et al. 2017), and the frailty syndrome involving the role of the dopaminergic system (Seiffert et al. 2017).

Ageing process is plastic and can be positively modulated by a proper life style, which refers mainly to moderate physical activity and healthy diet including supplementation with natural compounds. This topic is addressed by several review articles and research papers in this special issue. For example, Rea (2017) discusses that although many of the molecular pathways remain to be fully identified, physical activity and exercise is understood to produce changes in the human epigenome, which have the potential to enhance cognitive and psychological health, improve muscular fitness, and lead to better ageing with improved quality of life in older age. The beneficial effects of Nordic Walking (NW) is presented in two original papers. Kortas et al. (2017) show that NW reduced body iron stores and it was associated with lower oxidative stress and better endurance and Gmiat et al. (2017) show the beneficial influence of NW for some pro-inflammatory parameters. Physical activity also reduces visceral fat area and lowers the risk of metabolic syndrome (Zajac-Gawlak et al. 2017).

In the reviews by Grabowska et al. (2017) and Cavinato et al. (2017) information of positive role of natural compounds can be found. The first review concentrates on the role of the “proteins of youth”—sirtuins in ageing and longevity and how their modulation by natural compounds can prolong health- and life-span of model organisms. The second one acquainted us with the updated knowledge concerning plant extracts and natural compounds that are able to protect or mitigate the deleterious effects caused by photoaging in different experimental models.

Jagota and Mattam (2017) present the results of their studies on the some beneficial effect of melatonin on the daily chronomics of proteomic profiles of ageing and rotenone-induced Parkinsonism in rats Interestingly, intermittent fasting or dietary restriction is an increasingly popular intervention to promote healthy ageing and to delay age-associated decline in brain functions. Studies by Singh et al. (2017) demonstrated that the intermediate fasting regimen with herbal supplementation reduced anxiety-like behavior and associated neuroinflammation in middle aged female rats by ameliorating key inflammatory cytokines and modulated stress response. Dietary restriction is an example of mild stress-induced hormesis, and so Kyriazis (2017), in his opinion article, addresses the issue of steadily increasing technology which has a widespread effect on health and ageing. He considers the possibility that humans are subjected to beneficial hormetic effects of machines, which upregulate the stress response and modulate adaptation.

It seems that the field of biogerontology is undergoing rapid changes and developments. Although there is still a lot of “more of the same” going on in terms of testing individual natural and synthetic molecules in model systems, there is also an increasing awareness that if we really want to make breakthrough achievements in promoting health in old age, we must incorporate wholistic approaches such as hormesis, life-style variations, daily rhythms, and social interactions. The future of ageing is hopeful and promising.
